# Nurse-led early mobilization and ICU-acquired weakness in mechanically ventilated adults: a retrospective before-after cohort study

**DOI:** 10.3389/fmed.2026.1919355

**Published:** 2026-09-17

**Authors:** Haiyun Yang, Aijuan Zhang, Gaigai Nie, Weijie Ye

**Affiliations:** Department of Critical Care Medicine, Suzhou Wuzhong People’s Hospital, Suzhou, Jiangsu, China

**Keywords:** artificial respiration, critical illness, delirium, early ambulation, ICU-acquired weakness, intensive care units, muscle weakness, nursing care

## Abstract

**Introduction:**

ICU-acquired weakness (ICU-AW) is a frequent complication of critical illness, and real-world evidence on nurse-led early mobilization (EM) in Asian ICUs remains limited.

**Methods:**

We conducted a single-center, retrospective before-after cohort study of invasively ventilated adults admitted during usual-care (*n* = 153) and nurse-led EM (*n* = 164) periods. ICU-AW, defined as a Medical Research Council (MRC) sum score <48, was assessed among evaluable ICU survivors (*n* = 266); ICU death made volitional strength testing non-evaluable and was addressed in a death-or-ICU-AW composite sensitivity analysis. The primary model estimated the association between protocol implementation and ICU-AW after adjustment for baseline confounders; post-exposure care processes were examined separately.

**Results:**

ICU-AW occurred in 60/129 (46.5%) usual-care survivors and 40/137 (29.2%) nurse-led EM survivors (crude absolute risk difference 17.3 percentage points [95% CI 5.8–28.8] among evaluable survivors; crude OR 0.474, 95% CI 0.286–0.786). The baseline-adjusted OR was 0.436 (95% CI 0.248–0.766; *P* = 0.004). Morbidity-related secondary outcomes favored nurse-led EM, including lower delirium incidence (41.2% vs. 22.6%; OR 0.416), shorter ICU stay (median 11 vs. 8 days), and higher discharge Barthel Index (70 vs. 85). No statistically significant ICU mortality difference was detected (15.7% vs. 16.5%; OR 1.059, 95% CI 0.581–1.93), but the study was not powered to exclude clinically important mortality effects.

**Discussion:**

Nurse-led EM implementation was associated with lower odds of ICU-AW and improved morbidity outcomes. Because exposure was assigned by historical period, residual and secular confounding remain important limitations, and confirmation in a randomized or stepped-wedge implementation study is warranted.

## 1 Introduction

Intensive care unit-acquired weakness (ICU-AW) is a common and clinically consequential neuromuscular complication of critical illness, characterized by symmetrical, generalized limb muscle weakness arising during the ICU stay for which no cause other than the acute illness and its management can be identified (1). According to the American Thoracic Society clinical practice guideline, the bedside reference standard for diagnosis is volitional strength testing using the Medical Research Council (MRC) sum-score across six muscle groups bilaterally, with a total score below 48 (of a maximum of 60) indicating clinically significant weakness in an awake, cooperative patient (2). Reported incidence among invasively ventilated adults varies widely across case mix and ascertainment methods, with systematic review and prospective cohort data showing high rates in prolonged critical illness and mechanically ventilated populations (3, 4).

The pathophysiology of ICU-AW is multifactorial, driven by systemic inflammation, microvascular dysfunction, bioenergetic failure, and accelerated muscle proteolysis (5). Prolonged immobility and disuse atrophy further compound these processes, producing rapid loss of muscle mass and contractile function within the first days of critical illness (6). Critical illness polyneuropathy and myopathy are major clinical substrates of this syndrome (7). The consequences are substantial and durable, including prolonged mechanical ventilation, extended ICU and hospital length of stay, impaired functional recovery, and reduced long-term quality of life and survival (8, 9). Established risk factors include sepsis and systemic inflammation, illness severity, hyperglycemia, corticosteroid and neuromuscular blocking agent exposure, immobilization, deep sedation, and the use of physical restraint (10).

Early mobilization (EM) has emerged as a leading modifiable strategy to mitigate immobility-related harm, supported by randomized trials, quality-improvement work, safety consensus recommendations, and systematic reviews of ICU physical therapy and active mobilization (11–16). However, the evidence base remains heterogeneous: a large pragmatic trial of increased early mobilization found no survival benefit and raised concerns about aggressive dose escalation (17), while randomized trials of standardized rehabilitation or cycling/electrical stimulation have shown mixed effects on length of stay and muscle strength (18, 19); a later nursing-focused meta-analysis also found outcome heterogeneity across protocols and populations (20). EM is also aligned with ICU liberation and PADIS guidance through coordinated attention to pain, sedation, delirium, immobility, and sleep (21–23). Because bedside nurses provide continuous patient surveillance and coordinate sedation, delirium screening, and daily activity, nurse-led EM protocols are an attractive, scalable model for embedding mobilization into routine practice without dependence on scarce rehabilitation staffing. Critically, real-world effectiveness data from Asian ICU cohorts, where staffing models, sedation practices, and rehabilitation resources differ from those in the predominantly Western trial settings, remain scarce, constituting an important evidence gap. A recent umbrella review of systematic reviews and meta-analyses concluded that early rehabilitation significantly reduces the incidence of ICU-AW (risk difference −0.10; 95% CI −0.18 to −0.03), with greater benefit when rehabilitation is initiated within the first 1–3 days of ICU admission, while emphasizing persistent uncertainty about the optimal type, timing, and dose of rehabilitation and a paucity of nurse-led, real-world implementation data (24).

Interpretation of ICU-AW outcomes is further complicated by methodological challenges that prior observational work has not consistently addressed. Observational before-after analyses benefit from explicit causal framing of the target exposure, eligibility window, time zero, and estimand (25). Several care processes plausibly influenced by an EM protocol – reduced deep sedation, less physical restraint, and shorter ventilation – lie on the causal pathway and act as mediators rather than confounders, so conditioning on them changes the estimand from the total effect of protocol implementation to a controlled direct effect and requires stronger assumptions (26). Volitional strength can only be ascertained in evaluable survivors who are awake and cooperative; patients who die in the ICU are non-evaluable and represent a competing event rather than missing data, and conflating these mechanisms can bias effect estimates (27).

We therefore conducted a single-center retrospective before-after evaluation of a nurse-led EM protocol in a comprehensive adult ICU within an Asian healthcare setting, with explicit attention to these issues. The primary objective was to estimate the association between protocol implementation and ICU-AW among evaluable survivors, adjusting only for pre-exposure baseline confounders while recognizing ICU death as a non-evaluable competing outcome and treating intervening care processes as mediators in separate analyses. Secondary objectives examined strength, delirium, ventilation, length of stay, functional independence, and mortality. Recognizing the observational design, we hypothesized that nurse-led EM would be associated with a lower incidence of ICU-AW and improved secondary morbidity outcomes, without a detectable ICU mortality difference.

## 2 Materials and methods

### 2.1 Study design and setting

We conducted a single-center, retrospective before-after cohort study in a comprehensive adult intensive care unit (ICU) of Suzhou Wuzhong People’s Hospital, Suzhou, Jiangsu, China. The study compared two sequential, non-overlapping epochs: a pre-implementation period during which patients received usual care, and a post-implementation period during which a nurse-led early mobilization (EM) protocol was the unit standard of care. Reporting was aligned with STROBE guidance for observational studies (28). Because exposure was assigned by calendar epoch rather than by randomization or a concurrent control unit, group allocation may be confounded by secular trends, co-interventions and unmeasured temporal changes in case-mix or unit practice. Effect estimates should therefore be interpreted as associations of protocol implementation with outcomes under an observational before-after design; residual and secular confounding cannot be excluded. The pre-implementation (usual care) period spanned 1 June 2024 to 31 May 2025 and the post-implementation (nurse-led EM) period spanned 1 June 2025 to 31 May 2026; the two 12-month periods were consecutive, with no washout interval. Implementation of the nurse-led EM protocol was the only structured change introduced to unit practice as part of this study. The nurse-led EM protocol was introduced on 1 June 2025 as a routine clinical-care and nursing quality-improvement initiative, decided and implemented by the ICU clinical leadership independently of, and prior to the conception of, the present study; it was not an experimental intervention assigned for research purposes. The present retrospective analysis was subsequently approved by the Ethics Committee of Suzhou Wuzhong People’s Hospital (approval number 20261w10) on 23 June 2026, with a waiver of informed consent owing to the retrospective design. No identifiable, patient-level research data were accessed, extracted, or analyzed before this approval date; extraction of data from the electronic medical record and all analyses for the present study were undertaken only after ethics approval was granted.

### 2.2 Participants

Eligible patients were invasively mechanically ventilated adults (aged ≥18 years) admitted to the study ICU during the respective epochs. The pre-implementation (usual care) cohort comprised 153 patients and the post-implementation (nurse-led EM) cohort comprised 164 patients, for a total of 317 patients. During the two epochs, 356 consecutively admitted invasively ventilated adults were screened for eligibility (172 in the usual-care period and 184 in the nurse-led EM period). The participant-level time origin (day 0) was defined as the first calendar day of invasive mechanical ventilation in the study ICU. Eligibility required invasive mechanical ventilation for at least 48 h; for patients with more than one ICU admission during a study period, only the first qualifying admission was included, and repeat admissions were not counted as separate observations. Pre-specified entry exclusion criteria, applied identically in both periods, were: pre-existing neuromuscular disease or another condition precluding valid volitional strength testing (e.g., Guillain–Barré syndrome, myasthenia gravis, motor neuron disease, or documented pre-admission limb weakness); acute primary neurological injury with focal deficits (ischemic or hemorrhagic stroke, traumatic brain injury, or spinal cord injury); severe pre-admission functional dependence (baseline modified Rankin Scale ≥4); invasive ventilation for less than 48 h; limb amputation or injury preventing bilateral MRC testing; and an ICU stay too brief to permit protocol exposure or outcome ascertainment. After applying these criteria, 39 patients (19 usual care, 20 nurse-led EM) were excluded at entry, leaving 153 and 164 eligible patients, respectively (total 317). Each patient was followed from day 0 to ICU discharge or death, with in-hospital outcomes tracked to hospital discharge; patients transferred to another ICU or facility before ICU discharge were followed to the point of transfer and, if strength could not be assessed before transfer, were classified as non-evaluable for the primary outcome. Because volitional strength testing requires an awake, cooperative patient, 51 patients who died in the ICU (24 usual care, 27 nurse-led EM) were non-evaluable for ICU-AW, leaving 266 evaluable survivors (129 usual care, 137 nurse-led EM) for the primary analysis. ICU death was the only reason for non-evaluability in this cohort: all 266 ICU survivors regained an awake, cooperative state permitting volitional MRC testing before ICU discharge, none were transferred before strength assessment, and there were consequently no missing primary-outcome data among survivors. Patients excluded at entry (*n* = 39) are thus distinct from patients who were included but non-evaluable for the primary outcome (*n* = 51, all ICU deaths). The participant flow is shown in Figure 1.

**FIGURE 1:**
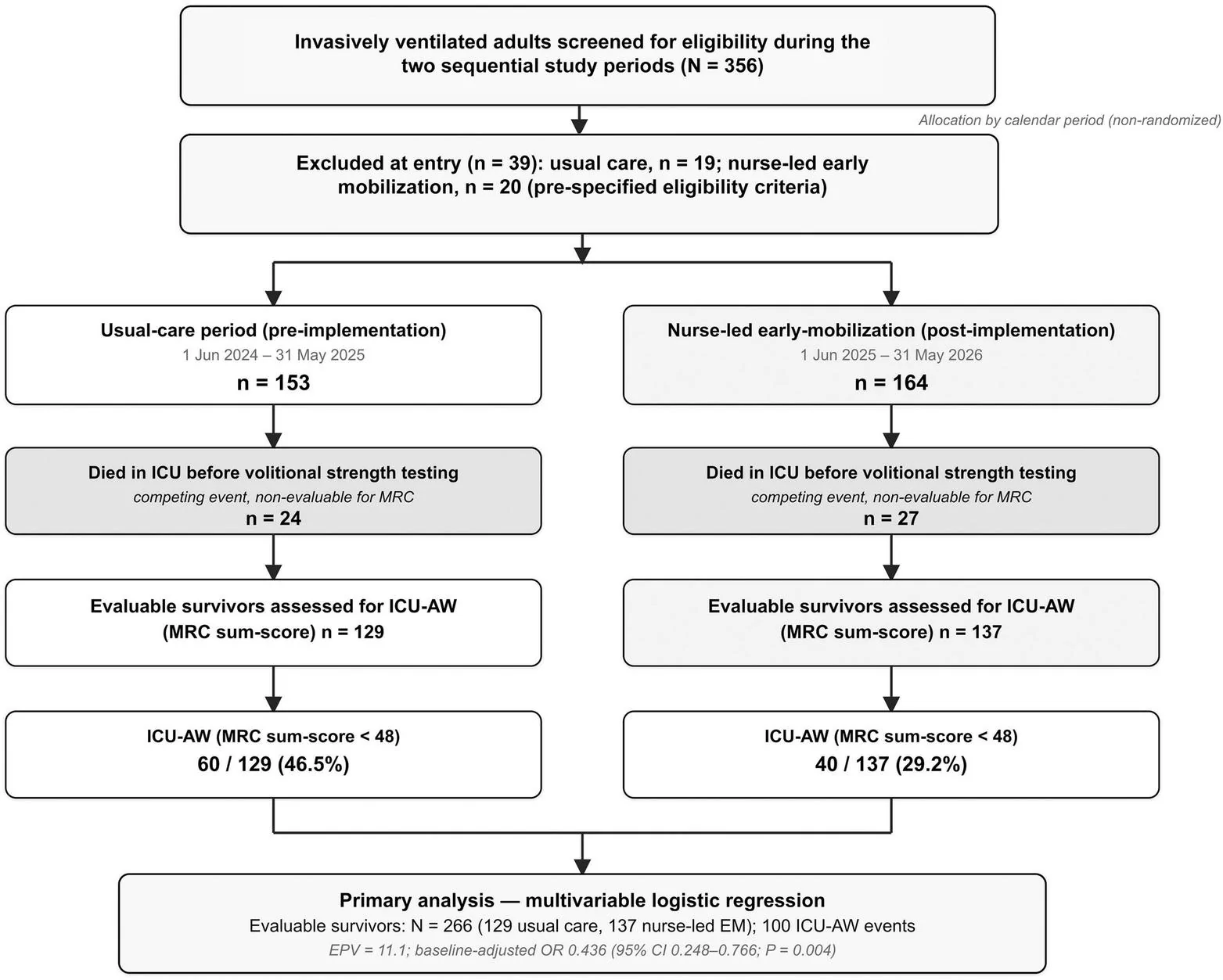
STROBE participant flow diagram.

### 2.3 The nurse-led early mobilization intervention

The intervention was a nurse-driven, protocolized EM bundle. Bedside nurses performed structured daily screening for mobilization readiness using predefined domain-based safety criteria consistent with published ICU mobilization recommendations. Readiness screening covered hemodynamic stability (no untreated shock, unstable arrhythmia, or rapidly escalating vasoactive support), respiratory stability (adequate oxygenation without new respiratory distress or unsafe ventilator settings), neurological readiness (ability to participate in or tolerate passive/assisted activity without dangerous agitation), and device security (airway, vascular catheters, drains, and monitoring lines secured before activity). Mobilization was deferred, stepped down, or stopped for new hemodynamic instability, oxygen desaturation, respiratory distress, reduced consciousness, unsafe agitation, uncontrolled pain, active bleeding, or concern for tube or line dislodgement. Eligible patients were advanced through a progressive mobility ladder (passive and active range of motion, sitting, standing, and ambulation as tolerated). Routine nursing documentation captured readiness screening, achieved mobility level, and active mobilization minutes. The protocol targeted first mobilization within approximately 2 days of intubation (median 2 days) and a daily active mobilization dose of approximately 21 min per day (median), contrasting with usual care in which first mobilization occurred later (median approximately 5 days) and the daily dose was lower (median approximately 7 min per day). Daily active mobilization dose was calculated for each patient as the total documented active mobilization minutes accrued during the ICU stay divided by the number of ICU days on which the patient was alive and eligible for mobilization (ICU days from day 0 to ICU discharge or death), yielding a per-patient average of minutes per day; days on which mobilization was contraindicated by the readiness criteria were retained in the denominator. Because this metric is derived entirely from care delivered after protocol exposure and accrues only over days survived in the ICU, it is susceptible to opportunity and immortal-time bias and is treated throughout as a descriptive, exploratory exposure rather than a randomized or independently assigned dose (Section “2.7 Statistical analysis”).

### 2.4 Usual care

In the pre-implementation epoch, mobilization was delivered at the discretion of the treating team without a standardized nurse-led screening or escalation protocol, reflecting prevailing unit practice.

### 2.5 Outcomes

The primary outcome was ICU-acquired weakness (ICU-AW), defined according to American Thoracic Society 2014 criteria as an MRC sum score below 48 (2). Because volitional strength testing requires an awake, cooperative patient, ICU-AW was ascertained only in evaluable ICU survivors (*n* = 266). MRC sum-score and ICU-AW were ascertained by trained assessors once patients were awake and able to follow commands, consistent with the American Thoracic Society requirement for volitional strength testing, at or before ICU discharge and applying identical criteria in both periods. MRC sum-score testing was performed and documented prospectively as part of routine bedside nursing and rehabilitation care in both study periods using a standardized institutional procedure (bilateral testing of three upper-limb and three lower-limb muscle groups, each graded 0–5 and summed to a 0–60 score); scores were recorded contemporaneously in the electronic medical record and were not reconstructed retrospectively for this analysis. Assessments were conducted by trained ICU nurses and physiotherapists who had completed structured competency training in MRC manual muscle testing; each patient’s strength was scored independently by two assessors, and inter-rater agreement in a random 15% subsample was high (weighted kappa 0.86; intraclass correlation coefficient 0.91). The first valid assessment obtained once the patient was awake and able to follow commands (Richmond Agitation-Sedation Scale −1 to +1 and able to complete a five-item command examination), at or before ICU discharge, was used. The median time from ICU admission to MRC assessment was similar in the two periods (usual care 9 days [IQR 6–13] vs. nurse-led EM 8 days [IQR 5–11]). Because assessments were embedded in routine care, assessors were not blinded to study period or exposure status, and the possibility of recovery-time bias, whereby patients who awaken and cooperate earlier are assessed at a different point in their illness trajectory, cannot be excluded. Reasons for non-evaluability were limited to ICU death (*n* = 51); no survivor was non-evaluable owing to persistent coma, ongoing sedation, or transfer. Because ascertainment required an awake, cooperative patient, the timing of assessment was anchored to clinical recovery rather than fixed at a predetermined ICU day. ICU deaths (*n* = 51) made the volitional strength endpoint non-evaluable rather than missing at random; MRC sum score, handgrip strength, ICU-AW status and discharge Barthel Index were therefore missing by design for these patients. Robustness to this non-evaluable outcome was assessed using a death-or-ICU-AW composite sensitivity analysis. Secondary outcomes were MRC sum score, handgrip strength, ICU delirium incidence (with delirium duration among affected patients reported descriptively), invasive mechanical ventilation (MV) duration, ventilator-free days at 28 days, ICU and hospital length of stay (LOS), discharge Barthel Index (survivors only), and ICU mortality. Ventilator-free days at 28 days were coded as 0 for patients who died before day 28 or remained invasively ventilated for 28 days or longer; otherwise, ventilator-free days were calculated as 28 minus the number of invasive MV days through day 28. Mobilization-related adverse events (e.g., falls, accidental extubation, unplanned catheter or tube removal, or hemodynamic/oxygenation instability requiring interruption of a session) were not collected as a structured or prospectively adjudicated endpoint and could not be reliably reconstructed from the retrospective records; comparative safety between groups was therefore not assessed.

### 2.6 Variables and the confounder-versus-mediator distinction

Consistent with the total-effect estimand (25, 26), we distinguished baseline confounders from post-exposure care processes. Pre-exposure baseline confounders (age, sex, APACHE II, SOFA, sepsis present at ICU admission (defined by Sepsis-3 criteria), chronic obstructive pulmonary disease (COPD), cardiovascular disease and chronic kidney disease) were eligible for adjustment in the primary model. By contrast, deep sedation, physical restraint, MV duration and mobilization dose may lie on the hypothesized causal pathway between the protocol and ICU-AW. Adjusting for these variables in the primary model would change the estimand by removing part of the indirect pathway and could also introduce bias if mediator-outcome common causes were unmeasured. These post-exposure variables were therefore excluded from the primary model and examined separately in an exploratory controlled-direct-effect analysis. Invasive MV duration is reported descriptively as a secondary outcome (Table 1) and is additionally treated as a potential mediator in the controlled-direct-effect analysis; it was not entered in the primary model. Pharmacologic co-treatments administered during the ICU stay (corticosteroids, neuromuscular blocking agents, aminoglycosides) and hyperglycemia are recognized ICU-AW risk factors but are partly determined after exposure to the protocol and may act as confounders, co-interventions, or mediators; they were therefore not included in the primary model and were instead examined in a sensitivity analysis (Section “2.7 Statistical analysis”).

**TABLE 1:** Primary and secondary outcomes (Holm-adjusted P for secondary outcomes).

Outcome	Usual care	Nurse-led EM	Effect estimate	*P*	P (Holm)
Primary outcome (evaluable ICU survivors, n = 266)					
ICU-acquired weakness, n/N (%)	60/129 (46.5%)	40/137 (29.2%)	OR 0.474 (0.286–0.786)	0.005	–
Secondary outcomes					
MRC sum score	50.0 (39.0–56.0)	54.0 (45.0–57.0)	HL shift 3.0	0.001	0.006
Handgrip strength, kg	14.9 (11.2–18.3)	16.8 (13.7–19.6)	HL shift 1.8	0.002	0.007
ICU delirium	63 (41.2%)	37 (22.6%)	OR 0.416 (0.256–0.678)	<0.001	0.003
Invasive MV duration, days	7.0 (5.0–10.0)	6.0 (4.0–9.0)	HL shift -1.0	<0.001	0.002
Ventilator-free days at 28 d	20.0 (15.0–23.0)	21.0 (16.8–24.0)	HL shift 1.0	0.021	0.043
ICU length of stay, days	11.0 (8.0–13.0)	8.0 (5.0–11.0)	HL shift -2.0	<0.001	<0.001
Hospital length of stay, days	21.0 (17.0–25.0)	19.0 (14.8–23.0)	HL shift -2.0	0.002	0.006
Barthel Index at discharge	70.0 (55.0–80.0)	85.0 (70.0–90.0)	HL shift 15.0	<0.001	<0.001
ICU mortality	24 (15.7%)	27 (16.5%)	OR 1.059 (0.581–1.93)	0.972	0.972

For Table 1, continuous effect estimates are Hodges–Lehmann shifts; odds ratios are shown with 95% CIs. Strength measures and Barthel Index were analyzed among evaluable ICU survivors; ICU deaths were non-evaluable for these endpoints by design. Ventilator-free days at 28 days were coded as 0 for patients who died before day 28 or remained invasively ventilated for 28 days or longer; otherwise, they were calculated as 28 minus invasive MV days through day 28. Delirium duration is reported descriptively among patients who developed delirium and was not included in the Holm-adjusted secondary-outcome family (nine outcomes); analyzing it over all patients would double-count delirium incidence.

### 2.7 Statistical analysis

Continuous variables were compared using Welch’s *t*-test when approximately normal and the Mann–Whitney *U* test with Hodges–Lehmann shift estimates otherwise; categorical variables were compared using the chi-square or Fisher’s exact test, as appropriate. Baseline balance was assessed with standardized mean differences (SMD). The primary analysis was a multivariable logistic regression estimating the association between protocol implementation and ICU-AW among evaluable survivors, adjusting only for the baseline confounders above (*n* = 266; 100 events; 9 predictors; events-per-variable [EPV] = 11.1). Model performance was summarized by McFadden pseudo-R^2^ (0.142), discrimination via the c-statistic (0.752), and calibration via the Hosmer–Lemeshow test (*P* = 0.93). Sensitivity analyses comprised: (i) a parsimonious model (intervention, age, APACHE II, sepsis); (ii) an exploratory controlled-direct-effect model additionally conditioning on deep sedation, physical restraint and MV duration; (iii) a death-or-ICU-AW composite outcome to assess robustness to non-evaluability from ICU death; and (iv) a model additionally adjusting for measured pharmacologic co-treatments (corticosteroids, neuromuscular blocking agents, aminoglycosides) and hyperglycemia. We further described ICU-AW incidence across quartiles of daily active mobilization using a linear trend test and a test for departure from linearity. Because daily dose was strongly correlated with group assignment (r = 0.70), this analysis was interpreted as descriptive rather than independent evidence of a biological dose-response relationship. Accordingly, the quartile dose analysis is reported strictly as exploratory and hypothesis-generating, and given its collinearity with study period and susceptibility to opportunity and immortal-time bias, no causal or dose-response inference is drawn from it. To control the family-wise error rate, *P*-values for the nine secondary outcomes were adjusted using the Holm procedure; the primary outcome is reported without multiplicity adjustment. All tests were two-sided with alpha = 0.05. Analyses were performed in Python (pandas, scipy, statsmodels). No *a priori* sample-size calculation was performed, because the study analyzed all consecutive eligible patients over two fixed, pre-defined 12-month epochs rather than a prospectively recruited sample; the sample size was therefore determined by unit throughput. To characterize the precision afforded by this convenience sample, we noted *post hoc* that the 266 evaluable survivors (129 usual care, 137 nurse-led EM) provided approximately 83% power at a two-sided alpha of 0.05 to detect the observed absolute reduction in ICU-AW from 46.5% to 29.2% (odds ratio approximately 0.47); detecting an absolute difference of comparable magnitude (46% vs. 30%) with 80% power would require approximately 144 evaluable patients per group. These figures describe achieved precision only and are not a substitute for a prospectively powered design; the widths of the reported 95% confidence intervals, particularly for mortality, should be used to judge the precision of each estimate.

## 3 Results

### 3.1 Study population and baseline comparability

A total of 317 invasively ventilated adults were analyzed (Figure 1): 153 received usual care before implementation and 164 received the nurse-led early-mobilization (EM) protocol after implementation. The two groups were well balanced across all measured pre-exposure characteristics (Table 2): age (60.3 ± 14.0 vs. 61.2 ± 14.6 years), APACHE II (median 21.0 [16.0–27.0] vs. 21.0 [15.0–24.0]), SOFA (7.0 [5.0–9.0] vs. 7.0 [6.0–9.0]), male sex (69.3% vs. 66.5%), and sepsis (33.3% vs. 37.8%). The sepsis variable used for baseline adjustment denotes sepsis present at ICU admission (day 0), defined by Sepsis-3 criteria, and therefore represents a pre-exposure baseline characteristic rather than sepsis arising later during the ICU stay. All standardized mean differences were small (absolute SMD ≤ 0.21, the largest being for cardiovascular disease), and no baseline comparison reached statistical significance (all P > 0.05; the closest, cardiovascular disease, P = 0.09). Measured ICU pharmacologic co-treatments were largely comparable, with a modestly higher use of corticosteroids in the EM period (28.8% vs. 37.8%; SMD 0.19) and balanced use of neuromuscular blocking agents (12.4% vs. 9.1%) and aminoglycosides (15.7% vs. 15.9%) (Table 2).

**TABLE 2:** Patient characteristics and post-exposure care processes by study group.

Characteristic	Usual care (*n* = 153)	Nurse-led EM (*n* = 164)	*P*	SMD
A: Pre-exposure baseline characteristics				
Age, years	60.3 ± 14.0	61.2 ± 14.6	0.559	−0.066
BMI, kg/m^2^	22.3 (20.1–24.3)	22.4 (20.6–24.5)	0.907	−0.024
APACHE II score	21.0 (16.0–27.0)	21.0 (15.0–24.0)	0.399	0.119
SOFA score	7.0 (5.0–9.0)	7.0 (6.0–9.0)	0.675	−0.077
B: ICU-stay measures and co-treatments				
Peak blood glucose, mmol/L	11.1 ± 3.3	11.2 ± 3.1	0.927	−0.01
Peak lactate, mmol/L	2.5 (1.5–3.6)	2.6 (1.5–3.8)	0.631	−0.028
Serum albumin (nadir), g/L	29.8 ± 5.7	30.0 ± 5.2	0.797	−0.029
Male sex	106 (69.3%)	109 (66.5%)	0.677	−0.06
Hypertension	54 (35.3%)	50 (30.5%)	0.429	−0.102
Diabetes mellitus	24 (15.7%)	33 (20.1%)	0.378	0.116
COPD	23 (15.0%)	19 (11.6%)	0.46	−0.102
Cardiovascular disease	23 (15.0%)	38 (23.2%)	0.09	0.208
Chronic kidney disease	17 (11.1%)	13 (7.9%)	0.438	−0.109
Admission diagnosis, n (%)			0.73	
Sepsis/infection	55 (35.9%)	50 (30.5%)		
Respiratory failure/pneumonia	27 (17.6%)	32 (19.5%)		
Postoperative	44 (28.8%)	46 (28.0%)		
Trauma	12 (7.8%)	19 (11.6%)		
Other	15 (9.8%)	17 (10.4%)		
Sepsis	51 (33.3%)	62 (37.8%)	0.476	0.094
Septic shock	28 (18.3%)	30 (18.3%)	1.0	−0.0
Hyperglycemia (> 10 mmol/L)	98 (64.1%)	102 (62.2%)	0.821	−0.039
Vasoactive agents	66 (43.1%)	84 (51.2%)	0.184	0.162
Renal replacement therapy	18 (11.8%)	11 (6.7%)	0.172	−0.175
Corticosteroids	44 (28.8%)	62 (37.8%)	0.113	0.193
Neuromuscular blocking agents	19 (12.4%)	15 (9.1%)	0.448	−0.106
Aminoglycosides	24 (15.7%)	26 (15.9%)	1.0	0.005
C. Protocol-related exposures (post-exposure care processes) by study group.				
**Protocol / exposure measure**	**Usual care**	**Nurse-led EM**	***P***	
Time to first mobilization, days	5.0 (4.0–8.0)	2.0 (1.0–3.0)	<0.001	
Daily active mobilization, min	7.0 (4.0–10.0)	21.0 (15.0–26.2)	<0.001	
Invasive MV duration, days	7.0 (5.0–10.0)	6.0 (4.0–9.0)	<0.001	
Deep sedation (RASS ≤ −3 ≥ 48 h)	87 (56.9%)	65 (39.6%)	0.003	
Physical restraint	91 (59.5%)	52 (31.7%)	<0.001	

Data are mean ± SD, median (IQR), or *n* (%). SMD, standardized mean difference. ‘Sepsis/infection’ under Admission diagnosis denotes the primary reason for ICU admission, whereas the separate ‘Sepsis’ row denotes sepsis present at ICU admission (day 0), defined by Sepsis-3 criteria; this admission (baseline) status is the variable used for adjustment in the primary model. Corticosteroids, neuromuscular blocking agents, and aminoglycosides are pharmacologic co-treatments administered during the ICU stay and were examined in a sensitivity analysis rather than the primary model. These pharmacologic co-treatments are not baseline characteristics; they are presented in a separate block, distinct from the pre-exposure baseline covariates. Invasive mechanical ventilation duration is likewise an outcome and possible mediator rather than a baseline or intervention variable, and is reported among the outcomes (Table 1) and in the exploratory controlled-direct-effect analysis, not as a baseline characteristic.

The protocol-related exposures, which were treated as post-exposure care processes rather than baseline confounders, differed substantially between groups (Table 2C). Compared with usual care, the EM group had shorter time to first mobilization (median 5.0 [4.0–8.0] vs. 2.0 [1.0–3.0] days, *P* < 0.001), greater daily active mobilization (7.0 [4.0–10.0] vs. 21.0 [15.0–26.2] min, *P* < 0.001), shorter invasive mechanical ventilation (MV) duration (7.0 [5.0–10.0] vs. 6.0 [4.0–9.0] days, *P* < 0.001), less deep sedation (56.9% vs. 39.6%, *P* = 0.003), and less physical restraint (59.5% vs. 31.7%, *P* < 0.001).

### 3.2 Primary outcome: ICU-acquired weakness

ICU-AW was ascertained only in evaluable ICU survivors (*n* = 266); 51 ICU deaths were non-evaluable for volitional strength testing. ICU-AW occurred in 60/129 (46.5%) of evaluable patients under usual care versus 40/137 (29.2%) under nurse-led EM (crude OR 0.474, 95% CI 0.286–0.786, *P* = 0.005), corresponding to a crude absolute risk difference of 17.3 percentage points (95% CI 5.8 to 28.8 percentage points) among evaluable survivors. In the baseline-adjusted logistic model, the adjusted OR (aOR) for nurse-led EM was 0.436 (95% CI 0.248–0.766, *P* = 0.004) (Figure 2, Table 3); the crude and adjusted estimates were concordant, indicating little confounding by the measured baseline covariates, though not by unmeasured or secular factors. Other baseline predictors of ICU-AW were age (aOR 1.055 per year, 95% CI 1.031–1.079, *P* < 0.001), APACHE II (aOR 1.055, 95% CI 1.015–1.097, *P* = 0.006), and sepsis (aOR 2.437, 95% CI 1.373–4.327, *P* = 0.002); male sex, SOFA, COPD, cardiovascular disease, and chronic kidney disease were not statistically significant (Figure 3). The model (*n* = 266, 100 events, EPV = 11.1, McFadden pseudo-R^2^ = 0.142) showed acceptable discrimination (c-statistic 0.752) and no evidence of poor calibration (Hosmer–Lemeshow *P* = 0.93).

**FIGURE 2:**
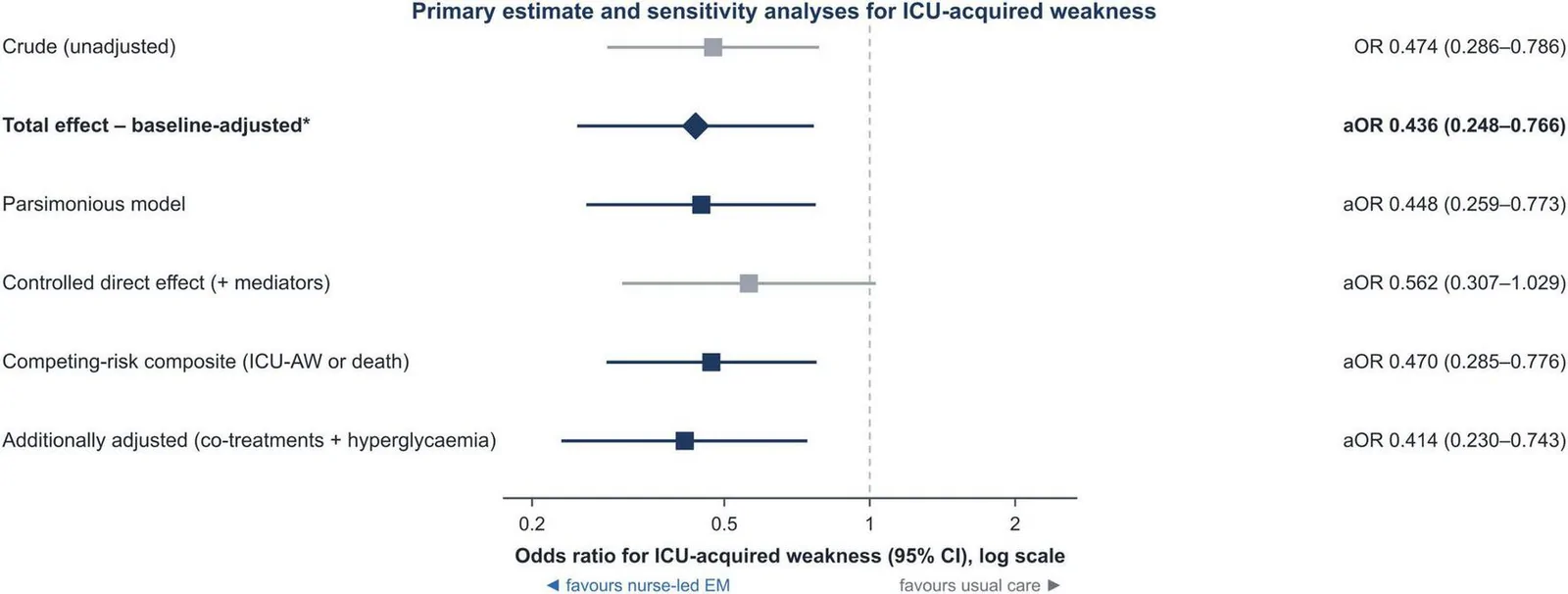
Primary outcome (ICU-AW): primary estimate and sensitivity analyses.

**TABLE 3:** Baseline-adjusted multivariable logistic regression for ICU-AW.

Variable	Adjusted OR	95% CI	P
Nurse-led early mobilization	0.436	0.248–0.766	0.004
Age, years	1.055	1.031–1.079	<0.001
Male sex	0.988	0.549–1.78	0.969
APACHE II score	1.055	1.015–1.097	0.006
SOFA score	1.073	0.975–1.18	0.148
Sepsis	2.437	1.373–4.327	0.002
COPD	0.496	0.214–1.148	0.102
Cardiovascular disease	0.637	0.304–1.335	0.233
Chronic kidney disease	0.667	0.245–1.81	0.426

Primary model: *n* = 266, events = 100, EPV = 11.11, McFadden pseudo-R^2^ = 0.142, c-statistic = 0.752, Hosmer–Lemeshow *P* = 0.928.

**FIGURE 3:**
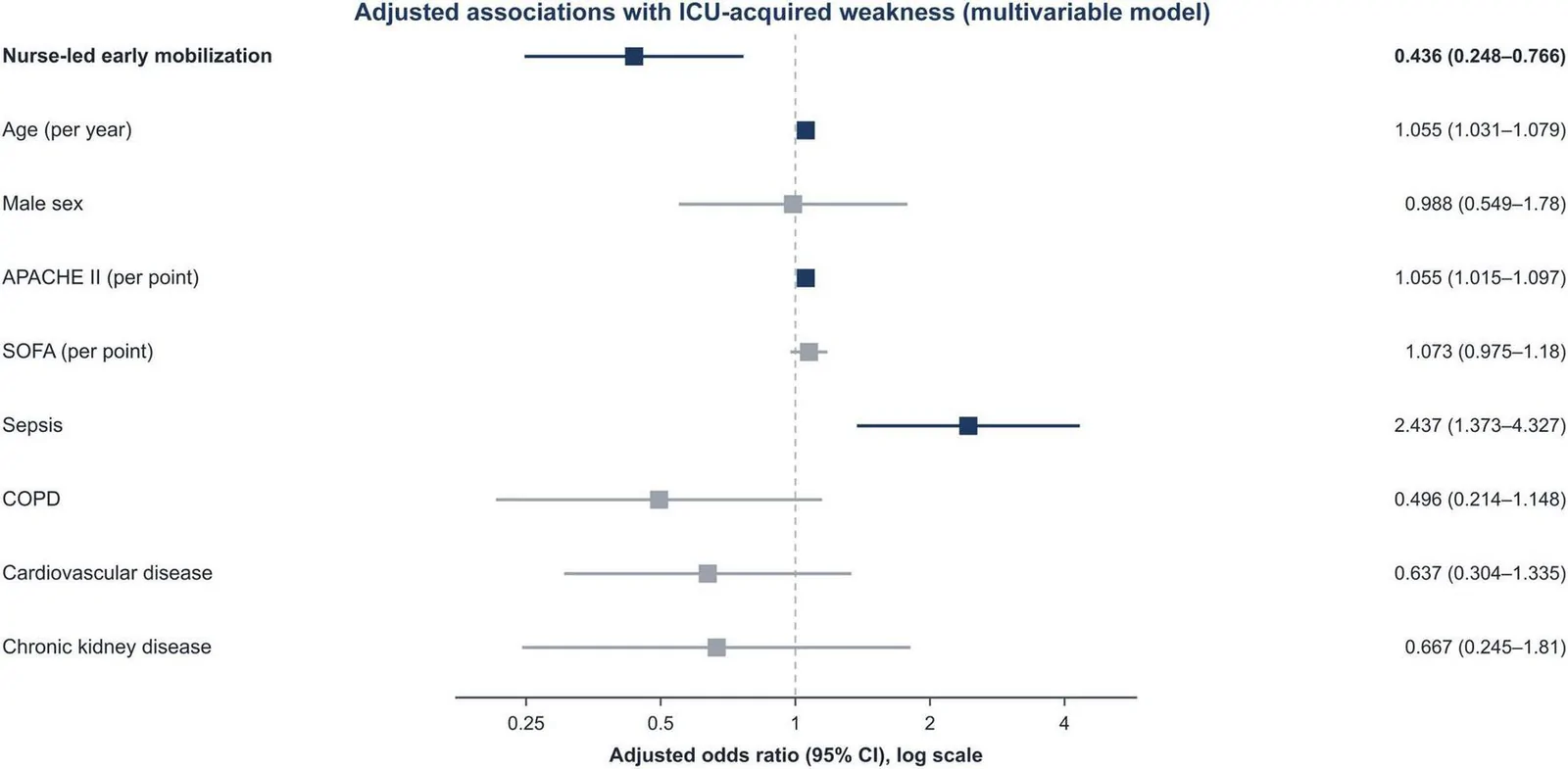
Multivariable model estimates for ICU-AW.

### 3.3 Secondary outcomes

Most morbidity-related secondary outcomes (Holm-adjusted across the nine secondary outcomes) favored nurse-led EM (Figure 4, Table 1). Strength measures (MRC sum score, handgrip) and the Barthel Index were assessed in survivors only and were missing by design for the 51 ICU deaths. The MRC sum score was higher (median 50.0 [39.0–56.0] vs. 54.0 [45.0–57.0], Holm *P* = 0.006), as was handgrip strength (14.9 vs. 16.8 kg, Holm *P* = 0.007). ICU delirium was less frequent (41.2% vs. 22.6%; OR 0.416, 95% CI 0.256–0.678, Holm *P* = 0.003). Among patients who developed delirium, duration was similar between groups (median 4.0 [IQR 3.0–5.0] days in each; *P* = 0.70), indicating that the lower delirium burden under nurse-led EM reflected a lower incidence rather than a shorter per-patient duration. Invasive MV duration was shorter (7.0 vs. 6.0 days, Holm *P* = 0.002) and ventilator-free days at 28 days were greater (20.0 [15.0–23.0] vs. 21.0 [16.8–24.0], Holm P = 0.043). ICU length of stay (11.0 vs. 8.0 days, Holm *P* < 0.001) and hospital length of stay (21.0 vs. 19.0 days, Holm P = 0.006) were both shorter. Among survivors, the Barthel Index at discharge was higher (70.0 [55.0–80.0] vs. 85.0 [70.0–90.0], Holm *P* < 0.001). No statistically significant ICU mortality difference was detected (15.7% vs. 16.5%; OR 1.059, 95% CI 0.581–1.93, P = 0.97). Structured mobilization-related adverse-event data were not available in the source records; comparative safety between the two periods could therefore not be assessed.

**FIGURE 4:**
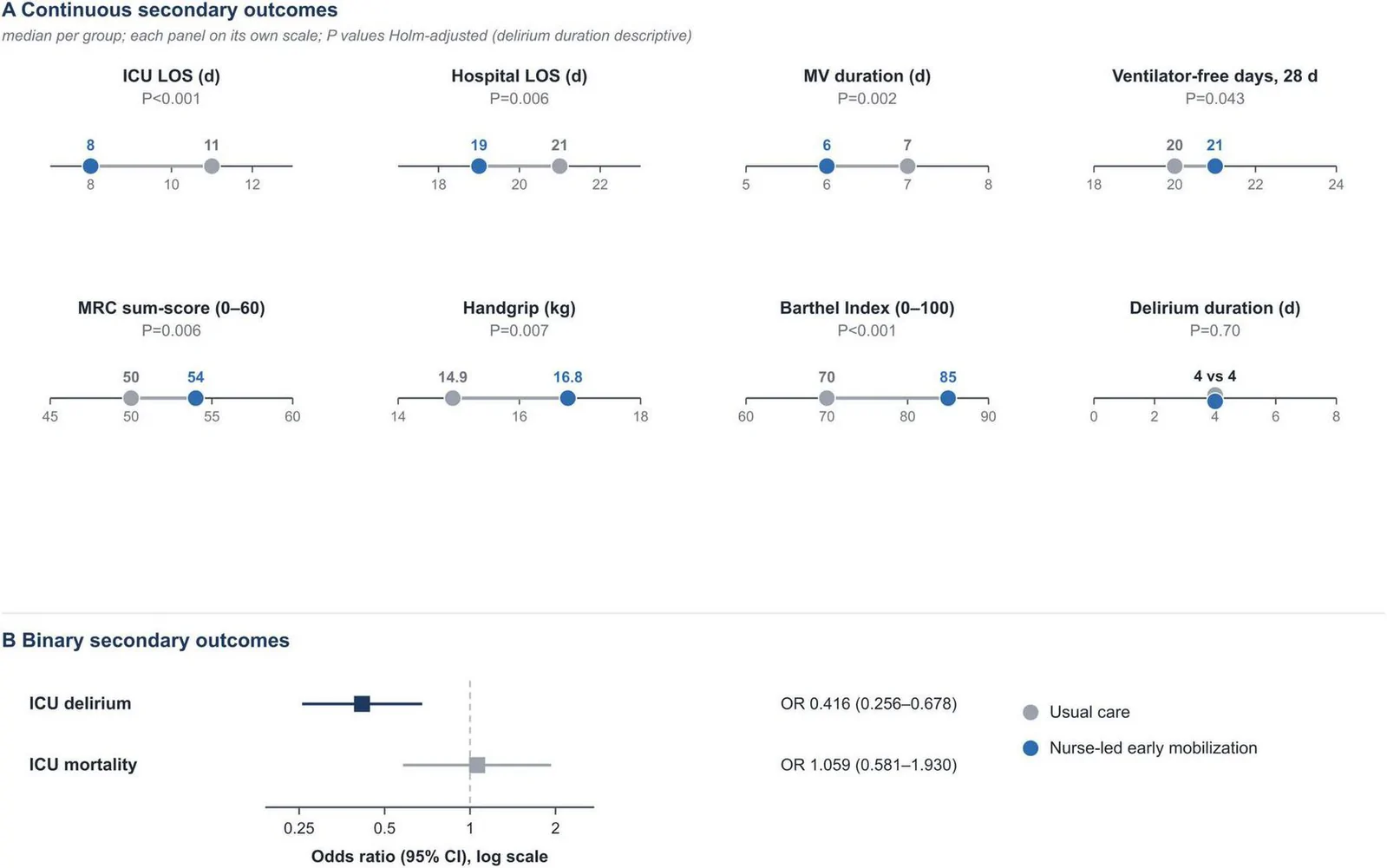
Secondary outcomes by study group (**A**, continuous small multiples; **B**, binary odds ratios).

### 3.4 Sensitivity and dose-response analyses

The primary estimate was similar across sensitivity analyses. A parsimonious model (intervention, age, APACHE II, sepsis) yielded aOR 0.448 (95% CI 0.259–0.773). An exploratory controlled-direct-effect model additionally conditioning on deep sedation, physical restraint and MV duration attenuated the estimate toward the null (aOR 0.562, 95% CI 0.307–1.029, *P* = 0.062), a pattern compatible with mediation through these care processes but not a formal decomposition of indirect and direct effects. The death-or-ICU-AW composite gave aOR 0.470 (95% CI 0.285–0.776, *P* = 0.003), supporting robustness to non-evaluability from ICU death. Additionally adjusting for measured pharmacologic co-treatments (corticosteroids, neuromuscular blocking agents, aminoglycosides) and hyperglycemia did not attenuate the association (aOR 0.414, 95% CI 0.230–0.743, *P* = 0.003); in that model hyperglycemia was independently associated with ICU-AW (aOR 2.14, 95% CI 1.14–4.02, *P* = 0.018) whereas the pharmacologic co-treatments were not. Because this model included 13 predictors for 100 events (EPV 7.7), it was interpreted as a secondary, exploratory analysis.

In a descriptive dose analysis, crude ICU-AW incidence decreased across quartiles of daily active mobilization (Q1 45.0%, Q2 43.4%, Q3 31.9%, Q4 29.7%; linear-trend *P* = 0.029; test for nonlinearity *P* = 0.95). However, this gradient provided no information independent of group assignment: daily dose was strongly correlated with group (*r* = 0.70), and when daily dose and group were entered together, the per-minute dose effect was null (aOR 0.995 per minute, *P* = 0.80) while the group effect persisted (aOR 0.48). The quartiles are therefore near-proxies for study period rather than independent within-group evidence of a graded effect.

## 4 Discussion

### 4.1 Principal findings

In this single-center retrospective before-after cohort of invasively ventilated adults, implementation of a nurse-led early mobilization (EM) protocol was associated with a lower occurrence of ICU-acquired weakness (ICU-AW) among evaluable survivors. The baseline-adjusted odds ratio was 0.436 (95% CI 0.248–0.766; *P* = 0.004), and the crude absolute risk difference among evaluable survivors was 17.3 percentage points (95% CI 5.8 to 28.8; 46.5% vs. 29.2%). Because these estimates come from nonrandomized historical cohorts and from patients evaluable for volitional strength testing, they should not be interpreted as a definitive causal effect size. The association was similar in the parsimonious model and in the death-or-ICU-AW composite sensitivity analysis. Morbidity-related secondary outcomes also favored nurse-led EM, including less delirium, shorter ICU and hospital stay, more ventilator-free days, and better discharge Barthel Index. No statistically significant ICU mortality difference was detected, but confidence intervals were too wide to exclude clinically important mortality effects.

### 4.2 Comparison with prior literature

These findings are consistent with the foundational trial of early physical and occupational therapy with interruption of sedation, which improved return to independent function and reduced ICU delirium (11); our study assessed related neuromuscular, cognitive, and functional domains rather than that trial’s identical functional endpoint. Our delirium reduction (41.2% to 22.6%; OR 0.416; 95% CI 0.256–0.678) and functional gains align with the broader morbidity signal in early-mobilization and rehabilitation literature (12–16, 20). The absence of a statistically significant mortality difference (15.7% vs. 16.5%; OR 1.06; 95% CI 0.58–1.93; *P* = 0.97) also aligns with prior evidence: a large multicenter trial of increased early mobilization found no survival benefit and signaled potential harms from aggressive dose escalation (17), randomized rehabilitation and cycling/electrical-stimulation trials reported mixed functional effects (18, 19), and systematic reviews reported no consistent mortality effect while supporting functional and process benefits (16, 20). Our results therefore add a nurse-led, protocolized implementation to a body of evidence in which benefits appear most plausible for morbidity-related outcomes rather than survival. Our findings are also concordant with a recent umbrella review of seven systematic reviews (69 primary studies), which reported that early rehabilitation reduces ICU-AW incidence (risk difference −0.10; 95% CI −0.18 to −0.03) and that earlier initiation (within 1–3 days) confers greater benefit, mirroring the early first-mobilization target (median 2 days) achieved under our nurse-led protocol (24). That review also identified the optimal rehabilitation modality, timing, and dose as unresolved and noted limited real-world and nurse-led implementation evidence, gaps that our single-center effectiveness analysis begins to address while reinforcing the need for the randomized or stepped-wedge confirmation we advocate.

### 4.3 Mechanistic interpretation: mediation and dose-response

The attenuation observed in the exploratory controlled-direct-effect model is compatible with, but does not prove or quantify, mediation through care processes (26). When deep sedation, physical restraint, and invasive ventilation duration were added to the model, the protocol estimate moved toward the null (aOR 0.562; 95% CI 0.307–1.029; *P* = 0.062). This pattern is expected if a nurse-led protocol acts partly by lightening sedation, reducing restraint, and bringing forward active mobilization, but formal mediation analysis would require strong additional assumptions and was not performed. The descriptive quartile analysis likewise should not be interpreted as an independent dose-response relationship because daily mobilization dose was highly collinear with study period. The associations of age (aOR 1.055 per year; 95% CI 1.031–1.079; *P* < 0.001), APACHE II (aOR 1.055; 95% CI 1.015–1.097; *P* = 0.006), and sepsis (aOR 2.437; 95% CI 1.373–4.327; *P* = 0.002) with ICU-AW are consistent with established ICU-AW risk-factor literature (10). Hyperglycemia was likewise independently associated with ICU-AW in a sensitivity analysis (aOR 2.14), and the protocol estimate was essentially unchanged after adjustment for measured pharmacologic co-treatments (corticosteroids, neuromuscular blocking agents, aminoglycosides), arguing against substantial confounding by these factors.

### 4.4 Clinical and nursing implications

The findings support further evaluation of a nurse-led, protocol-driven mobilization pathway as a feasible component of ICU care that may reduce neuromuscular and cognitive morbidity when paired with appropriate training, workflow support, safety screening, and interprofessional coordination. Nurses are continuously present at the bedside and are well positioned to coordinate sedation targets, restraint reduction, delirium screening, and daily activity progression. The observed reductions in ICU length of stay (median 11 to 8 days) and improved discharge Barthel Index (70 to 85) are outcomes of direct relevance to patients, families, and resource utilization, but they should be interpreted in light of the observational before-after design.

### 4.5 Limitations

Several limitations temper causal interpretation. First, the before-after design is vulnerable to secular trends and temporal confounding: co-occurring changes in sedation practice, staffing, rehabilitation resources, or unit culture could contribute to the observed differences, and residual confounding by unmeasured factors cannot be excluded. Second, this was a single-center study, limiting generalizability. Third, ICU-AW was ascertained only in evaluable survivors because volitional MRC strength testing cannot be scored in patients who die or remain non-cooperative; the death-or-ICU-AW composite supports robustness but does not eliminate selection concerns. Fourth, the dose analysis was strongly collinear with group assignment (*r* = 0.70), and formal mediation analysis was not performed. Fifth, MRC testing requires patient cooperation; ascertainment was therefore anchored to clinical recovery (awake and cooperative) using identical criteria in both periods rather than a fixed ICU day, although residual effects of assessment timing and effort cannot be fully excluded. Sixth, mobilization-related adverse events were not systematically captured in the retrospective records, so comparative safety could not be assessed; this is an important limitation, given that early mobilization of mechanically ventilated patients carries potential procedural risks (e.g., line or tube dislodgement, falls, and transient instability) that warrant prospective safety monitoring in future studies. Seventh, long-term functional follow-up was unavailable. Eighth, all outcomes, including the MRC-based primary outcome, were ascertained without blinding to study period, and because MRC testing depends on patient effort and cooperation, differential outcome ascertainment and assessor non-blinding may have biased the neuromuscular and functional estimates. Ninth, restricting the primary analysis to evaluable survivors introduces survivor-selection bias in addition to the competing-risk problem posed by ICU death; although no survivor in this cohort was non-evaluable for reasons other than death, non-death non-evaluability (persistent coma, delirium, or early transfer) is a recognized threat in other settings and, together with recovery-time bias, cannot be excluded as a source of residual bias. Tenth, several covariates and care processes were recorded over the ICU stay rather than strictly at a fixed pre-exposure instant, so the timing of some variables relative to exposure is uncertain, and any variable determined after protocol implementation may act as a mediator or collider rather than a baseline confounder. Eleventh, no validated baseline functional status (e.g., pre-admission Barthel Index) beyond the entry criterion was available, limiting adjustment for pre-illness disability. Twelfth, intervention fidelity was captured only through routine nursing documentation of readiness screening, achieved mobility level, and mobilization minutes; adherence to each protocol step, reasons for deferred sessions, and consistency of delivery were not independently audited, so intervention-fidelity data are incomplete. Thirteenth, ICU death was treated as a non-evaluable competing event for strength outcomes, but for duration outcomes, death shortens observed durations and can bias between-group comparisons; ventilator-free days at 28 days partly address this for ventilation, whereas ICU and hospital length of stay were not analyzed as formal competing-risk endpoints. Fourteenth, delirium was ascertained from routine documented screening rather than a research-protocolized instrument applied at fixed intervals, so delirium incidence and duration may be subject to between-period ascertainment differences. Finally, model diagnostics (EPV 11.1, c-statistic 0.752, Hosmer–Lemeshow *P* = 0.93) describe internal performance, not causal validity.

### 4.6 Future research

A multicenter, randomized or stepped-wedge implementation trial is needed to establish causal effect and to define the optimal mobilization dose and timing (29), ideally with formal, prospective mediation analysis quantifying the contributions of sedation, restraint, and ventilation duration (26). Longer-term endpoints – post-ICU physical function, cognition, and patient-reported quality of life – should be prioritized to determine whether in-ICU gains translate into durable recovery (30, 31).

## 5 Conclusion

In this retrospective before-after cohort, a nurse-led early mobilization protocol was associated with lower adjusted odds of ICU-acquired weakness among evaluable survivors (aOR 0.436; 95% CI 0.248–0.766), alongside lower delirium incidence, shorter length of stay, and better discharge function. No statistically significant ICU mortality difference was detected, although the study was not powered to exclude clinically important mortality effects. The attenuation observed after conditioning on post-exposure care processes was compatible with mediation but did not quantify indirect effects. Given the observational, single-center, before-after design, these associations require confirmation in a randomized or stepped-wedge implementation trial.

## Data Availability

The raw data supporting the conclusions of this article will be made available by the authors, without undue reservation.
